# Carbohydrate Metabolism and Carbon Fixation in *Roseobacter denitrificans* OCh114

**DOI:** 10.1371/journal.pone.0007233

**Published:** 2009-10-01

**Authors:** Kuo-Hsiang Tang, Xueyang Feng, Yinjie J. Tang, Robert E. Blankenship

**Affiliations:** 1 Departments of Biology and Chemistry, Washington University in St. Louis, St. Louis, Missouri, United States of America; 2 Department of Energy, Environment and Chemical Engineering, Washington University in St. Louis, St. Louis, Missouri, United States of America; National Institute on Aging, United States of America

## Abstract

The *Roseobacter* clade of aerobic marine proteobacteria, which compose 10–25% of the total marine bacterial community, has been reported to fix CO_2_, although it has not been determined what pathway is involved. In this study, we report the first metabolic studies on carbohydrate utilization, CO_2_ assimilation, and amino acid biosynthesis in the phototrophic *Roseobacter* clade bacterium *Roseobacter denitrificans* OCh114. We develop a new minimal medium containing defined carbon source(s), in which the requirements of yeast extract reported previously for the growth of *R. denitrificans* can be replaced by vitamin B_12_ (cyanocobalamin). Tracer experiments were carried out in *R. denitrificans* grown in a newly developed minimal medium containing isotopically labeled pyruvate, glucose or bicarbonate as a single carbon source or in combination. Through measurements of ^13^C-isotopomer labeling patterns in protein-derived amino acids, gene expression profiles, and enzymatic activity assays, we report that: (1) *R. denitrificans* uses the anaplerotic pathways mainly via the malic enzyme to fix 10–15% of protein carbon from CO_2_; (2) *R. denitrificans* employs the Entner-Doudoroff (ED) pathway for carbohydrate metabolism and the non-oxidative pentose phosphate pathway for the biosynthesis of histidine, ATP, and coenzymes; (3) the Embden-Meyerhof-Parnas (EMP, glycolysis) pathway is not active and the enzymatic activity of 6-phosphofructokinase (PFK) cannot be detected in *R. denitrificans*; and (4) isoleucine can be synthesized from both threonine-dependent (20% total flux) and citramalate-dependent (80% total flux) pathways using pyruvate as the sole carbon source.

## Introduction

Two of the most important sources of carbon sinks known in nature are absorption of CO_2_ by the oceans and photosynthesis by photosynthetic organisms. The marine *Roseobacter* clade are potentially major contributors to global CO_2_ fixation as they make up 10–25% of the total microbial community in some surface ocean ecosystems [1]–[4]. Some members of the *Roseobacter* clade belong to a group known as Aerobic Anoxygenic Phototrophs (AAPs), the only known organisms performing photosynthesis requiring oxygen but not producing oxygen, while other members are non-phototrophic. CO oxidation was confirmed experimentally for the non-phototrophic *Roseobacter* clade bacterium *Silicibacter pomeroyi* [5] and other *Roseobacter* clade [6], and CO_2_ fixation was suggested in several marine AAPs [7], while bioinformatic analysis in *Roseobacter* clade with completed genome sequence indicated that the genes encoding ribulose bisphosphate carboxylase/oxygenase (RUBISCO) and phosphoribulokinase required in the Calvin cycle for carbon fixation, as well as genes for other autotrophic CO_2_ fixation pathways, are missing in these bacteria [5], [8]–[10]. Thus, it has been of great interest to determine how *Roseobacter* clade bacteria can fix CO_2_, if they indeed fix CO_2_.

Anaplerotic pathways have been proposed as an alternative mechanism for CO_2_ fixation in *Roseobacter* clade [5], [8], [11], but have not been verified experimentally. Given that organisms in the *Roseobacter* clade are known to require organic carbon sources for growth [12], [13], understanding how *Roseobacter* clade bacteria utilize organic carbon and assimilate CO_2_ will help us understand the bio-energy metabolism, production of bioactive metabolites, and roles of global carbon cycle in these wide-spread marine bacteria.

Here, we report metabolic and biochemical studies of *Roseobacter denitrificans* OCh114 [14], which can denitrify, as its name indicates [13], and produce bacteriochlorophyll *a* (BChl *a*) aerobically. The genomic sequence of *R. denitrificans* [8] suggested that the tricarboxylic acid (TCA) cycle and anaplerotic pathways are complete, and that most of the genes for carbohydrate metabolism in the Embden-Meyerhof-Parnas (EMP, glycolysis), Entner-Doudoroff (ED), and pentose phosphate (PP) pathways are present and annotated. To understand the contributions of these enzymes and reaction pathways in the metabolism of *R. denitrificans*, we developed a minimal growth medium containing only defined carbon source(s), optimized the growth conditions of *R. denitrificans* in different defined carbon sources, and used isotopomer assisted metabolite analyses, biochemical approaches, and gene expression profiles to investigate the carbon assimilation, carbohydrate utilization, and amino acid biosynthesis pathways in *R. denitrificans*.

## Results

### Growth of R. denitrificans OCh114 in different growth conditions

**Figure 1:**
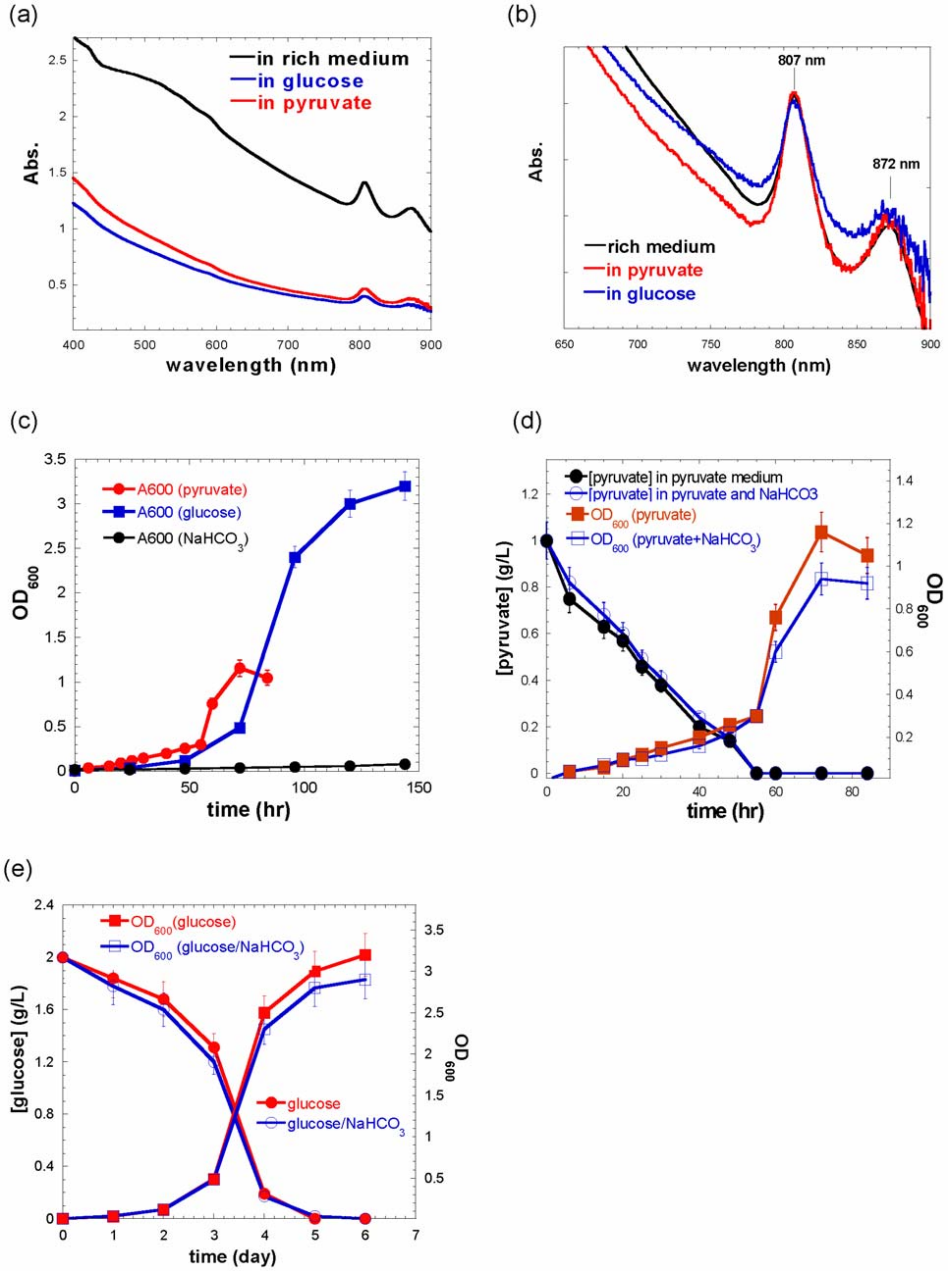
Spectra, image and organic carbon uptake of *R. denitrificans* OCh114. The spectra of cultures grown in the rich medium vs. in the minimal medium supplied with either 0.1% pyruvate or 0.1% glucose (a). No undefined carbon sources were included in the minimal medium reported in this paper. The normalized spectra in the 650 nm–900 nm range (b), the cell growth in the minimal medium with pyruvate, glucose, or HCO_3_^−^ (c), and the cell growth curve and uptake of pyruvate (d) or glucose (e) with or without the addition of 0.2% NaHCO_3_ in the minimal medium containing pyruvate or glucose. More than ten biological replicates were performed in every growth conditions.

Similar growth curves and spectral features were obtained in the *R. denitrificans* OCh114 cultures grown aerobically in the light (20 W/m^2^), dark, and day-night cycles (Figure S1a and S1b), and slightly higher OD_600_ were reached in the dark and day-light cycles compared to in the continuous light growth conditions (Figure S1c). It is consistent with the reports that light has a negative effect on pigment formation in some AAPs [15]. No growth was observed anaerobically regardless of the light intensity (data not shown). Multiple organic carbon sources have been tested for the growth of *R. denitrificans* OCh114 [13]. Here, we report studies of three carbon sources: pyruvate, D-glucose, and CO_2_ (or HCO_3_^−^). Spectral features of the photosynthesis system and light-harvesting complexes for the cultures grown in the minimal medium containing pyruvate or glucose are similar to the cultures grown in the rich medium (Figure 1a and 1b) as well as the results reported previously [13], [16]. The image of *R. denitrificans* cells grown in the minimal medium with pyruvate was examined by OLYMPUS FV1000/BX61 high-resolution confocal microscope, and the morphology and average cell size is consistent with previous reports [13]. Uptake of pyruvate, 2.5×10^−2^±5×10^−4^ mmole per hour (Figure 1d and S1d), is approximately 2 to 3-fold faster than uptake of glucose by *R. denitrificans* (Figure 1e), consistent with the faster growth in pyruvate than in D-glucose (Figure 1c). Higher OD_600_ and better cell growth can be obtained using 0.2% pyruvate in the minimal medium (data not shown). No differences in the ^13^C-isotopomer abundances of the protein-derived amino acids for cultures grown in the minimal medium containing pyruvate were observed under either dark or illuminated conditions (Table S1a and S1b). Similar results were also observed in the cultures grown in the minimal medium supplied with glucose.

### Vitamin B_12_ is required for the growth of R. denitrificans

The previous studies of the growth of *R. denitirficans* OCh114 or other AAPs have been performed in either a rich medium or a medium containing either 0.02 g/liter [13] or 0.1 g/liter [17] of yeast extract (undefined carbon sources). We confirmed the necessity of yeast extract, as poor growth of the *R. denitrificans* cultures without yeast extract was observed (data not shown). Although it has been recognized that yeast extract contains rich vitamin mixtures, it also includes amino acids and other undefined carbon sources. Ideally, the ^13^C-isotopic labeling studies require a minimal medium containing only defined carbon source(s), thus it is desirable to optimize the growth conditions by eliminating the yeast extract. We found that vitamin B_12_ (cyanocobalamin) can serve as an alternative to yeast extract for growing *R. denitrificans* in defined carbon sources, as cultures with OD_600_≥3 can be reached in our minimal growth medium with glucose as the sole carbon source (Figure 1e). Vitamin B_12_ and different forms of cobalamin are required for methionine and protein synthesis, deoxyribonucleotide triphosphate synthesis, amino acid metabolisms, and CO_2_ fixation (in methanogens, although there is no such pathway identified in *R. denitrificans*), and are included in the growth media of many photoautotrophic, photoheterotrophic and chemoheterotrophic bacteria. Requirement for vitamin B_12_ in the growth of *R. denitrificans* may partially explain why yeast extract was necessary to be included in the minimal medium containing either pyruvate or glucose.

### The carbohydrate utilization pathways in R. denitrificans

**Figure 2:**
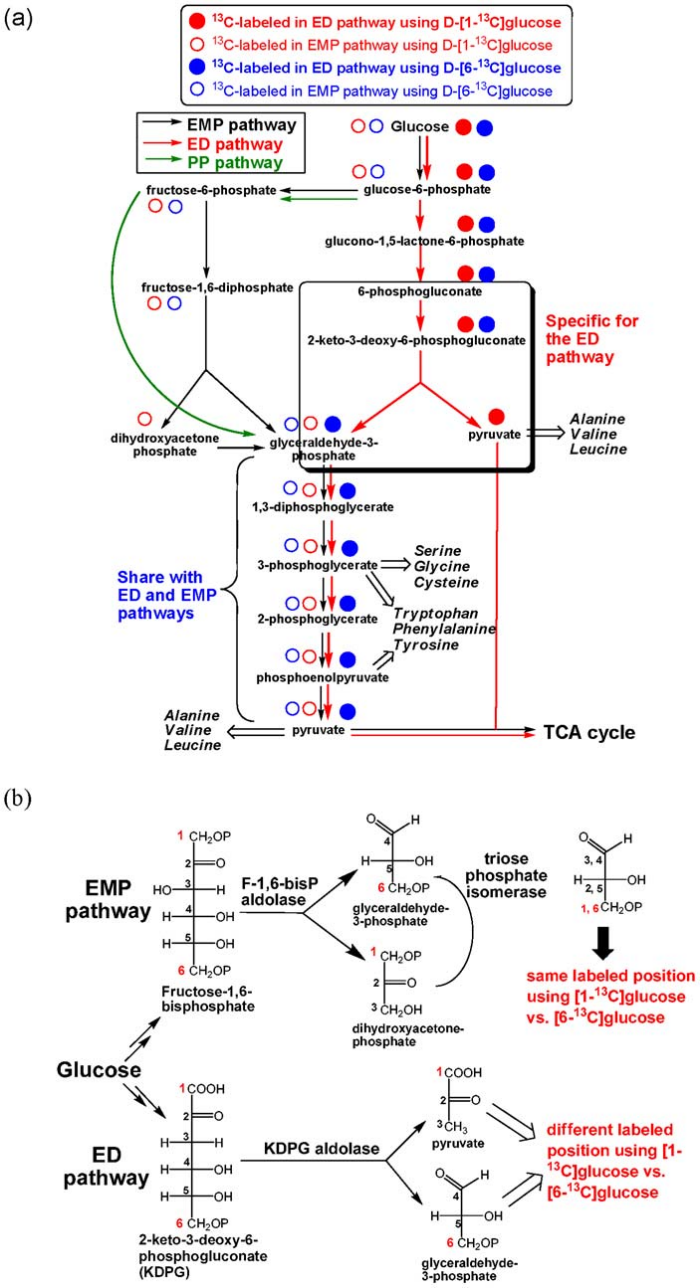
Proposed carbohydrate metabolism and amino acid biosynthesis pathways in *R. denitrificans*. Predicted ^13^C-labeling distributions using D-[1-^13^C]glucose and D-[6-^13^C]glucose in the EMP and ED pathways are shown (a), and the cleavage of a hexose molecule in the EMP vs. ED pathway (b). Abbreviations: EMP, Embden-Meyerhof-Parnas (glycolysis); ED, Entner-Doudoroff; and PP, pentose phosphate. Experimentally identified ^13^C-labeling patterns are reported in the context.

**Figure 3:**
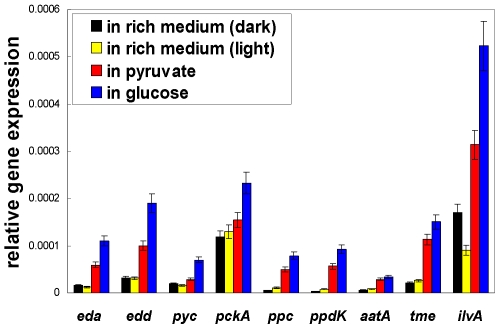
QRT-PCR studies. Gene name (encoding enzyme and gene location number): *eda* (2-keto-3-deoxy-6-phosphogluconate aldolase, RD1_2878), *edd* (6-phosphogluconate dehydratase, RD1_2879), *pyc* (pyruvate carboxylase, RD1_3376), *pckA* (phosphoenolpyruvate carboxykinase, RD1_1376), *ppc* (phosphoenolpyruvate carboxylase RD1_4248), *ppdK* (pyruvate phosphate dikinase, RD1_1948), *aatA* (aspirate aminotransferase, RD1_3892), *tme* (malic enzyme, RD1_0421) and *ilvA* (threonine deaminase, RD1_0416). Relative gene expression value of each gene is calculated with 2^−(ΔCt)^, where ΔCt = Ct_target gene_−Ct_16S rRNA gene_, and the 16S rRNA gene was used as the internal reference. Three biological replicates and eighteen technical replicates were preformed for every gene.

In the cultures grown in the minimal medium containing either D-[1-^13^C]glucose or D-[6-^13^C]glucose, the isotopomer labeling data of serine (the precursor is 3-phosphoglycerate) and alanine (the precursor is pyruvate) were different (Table S1e, S1f, S1i, and S3). Three metabolic pathways can be employed for sugar utilization by *R. denitrificans* and need to be considered to account for the isotopomer abundance in these protein-derived amino acids: (1) the Embden-Meyerhof-Parnas (EMP) pathway (glycolysis), by which one [1-^13^C]glucose molecule is cleaved into two glyceraldehyde-3-phosphate (GAP) molecules: one is [3-^13^C]-labeled and the other is unlabeled using either D-[1-^13^C]glucose or D-[6-^13^C]glucose; (2) the Entner-Doudoroff (ED) pathway [18], by which one molecule of glucose generates one molecule of GAP, in which the third carbon is labeled using D-[6-^13^C]glucose, and one molecule of pyruvate, where the first carbon is labeled using D-[1-^13^C]glucose (Figure 2a); (3) the pentose phosphate (PP) pathway, the first carbon of glucose is removed as CO_2_, if the oxidative PP pathway is active, to generate ribose-5-phosphate, which can be converted to GAP through the non-oxidative PP pathway (Figure S2). As shown in Figure 2a, regardless of the pathways, GAP is converted into 3-phosphoglycerate (3-PGA), the precursor of glycine, serine, and cysteine, and then to pyruvate, the precursor of alanine, valine, leucine, and isoleucine.

If *R. denitrificans* predominantly uses the EMP pathway for carbohydrate metabolism, one will expect similar ^13^C-isotopomer abundance in serine and alanine (converted from pyruvate through alanine aminotransferase). However, this is not consistent with the higher M+0 value (more unlabeled carbon) in serine (0.74) than in alanine (0.56) using D-[1-^13^C]glucose in our studies (Table S1e and S3). Alternatively, genes encoding two key enzymes in the ED pathway can be found in *R. denitrificans*: *eda* (RD1_2878), encoding 2-keto-3-deoxy-6-phosphogluconate (KDPG) aldolase (EC 4.1.2.14), and *edd* (RD1_2879), encoding phosphogluconate dehydratase (EC 4.2.1.12). If *R. denitrificans* uses the ED pathway as one of the alternative carbohydrate utilization pathways, [1-^13^C]glucose is converted into [1-^13^C]-2-keto-3-deoxy-6-phosphogluconate, which is cleaved into [1-^13^C]pyruvate and unlabeled GAP, leading to unlabeled 3-PGA (Figure 2b). In this case, serine is expected to be mostly unlabeled, while half of the alanine, derived from [1-^13^C] pyruvate, is labeled, consistent with our experimental data.

The QRT-PCR results indicate that both *eda* (RD1_2878) and *edd* (RD1_2879) genes are expressed and the transcript level of these genes is higher in the minimal medium containing either pyruvate or glucose than in the rich medium, similar to the gene expression profiles of other genes responsible for carbon fixation and carbon metabolism examined in this report (Figure 3). Moreover, the activity of 2-keto-3-deoxy-phosphogluconate (KDPG) aldolase and phosphogluconate dehydrase can be detected in cell-free extracts. Alternatively, the gene encoding 6-phosphofructokinase (PFK, EC 2.7.1.11), an essential enzyme for the EMP pathway, is not annotated, and no PFK activity can be detected in cell free extracts.

The proposed ED pathway was further tested using D-[6-^13^C]glucose, in which [6-^13^C]KDPG is cleaved into unlabeled pyruvate and [3-^13^C]GAP in the ED pathway (Figure 2b). Our data showed a smaller M+0 value in serine using D-[6-^13^C]glucose (0.30) than using D-[1-^13^C]glucose (0.74), compared to slightly higher M+0 value in alanine using D-[1-^13^C]glucose (0.56) versus D-[6-^13^C] glucose (0.48) (Table S1e and S1i). The M+0 value in serine using D-[1-^13^C]glucose or D-[6-^13^C]glucose is not close to 1 or 0, respectively, suggesting that in addition to the ED pathway, the PP pathway is also active and generating GAP with different labeling pattern using D-[6-^13^C]glucose or D-[1-^13^C] glucose (Figure 2a). Based on the isotopomer abundance of serine using D-[1-^13^C]glucose and D-[6-^13^C]glucose, we estimated 25∼30% of GAP is produced from the (non-oxidative) PP pathway (Table S3).

**Figure 4:**
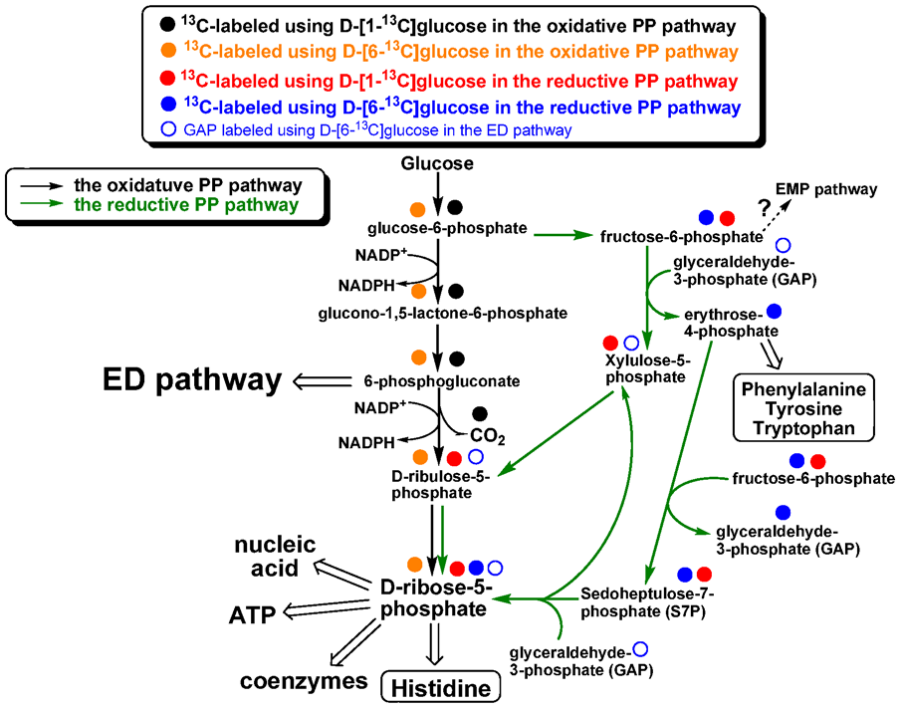
Proposed pentose phosphate pathway in *R. denitrificans*. All of the steps are reversible, except expulsion of CO_2_ catalyzed by 6-phosphogluconate dehydrogenase. Predicted ^13^C-labeling distributions using D-[1-^13^C]glucose and D-[6-^13^C]glucose in the oxidative PP and non-oxidative PP pathways are shown. Experimentally identified ^13^C-labeling patterns are reported in the context.

The ED pathway also leads to different predicted labeling in the aromatic amino acids. Phosphoenolpyruvate (PEP), synthesized from 3-PGA, and erythrose-4-phosphate (E4P), the intermediate in the non-oxidative PP pathway, are the precursors of phenylalanine, tyrosine and tryptophan. Labeled E4P in the non-oxidative PP pathway (Figure S2) leads to lower M+0 value in the aromatic amino acids (0.50±0.03) compared to serine (0.74) using D-[1-^13^C]glucose. The ED and PP pathways can explain much higher M+0 value in phenylalanine (0.48) and tyrosine (0.53) using D-[1-^13^C]glucose than the value in these amino acids (0.04) using D-[6-^13^C]glucose, and more labeled carbon can be incorporated into PEP and E4P using D-[6-^13^C]glucose (Figure 2a and 4).

### The Pentose Phosphate pathway in R. denitrificans

A smaller M+0 value in histidine (0.28) than other amino acids (0.42–0.89) was detected using D-[1-^13^C]glucose (Table S1e). Histidine is synthesized from 5-phospho-ribosyl-α-pyrophosphate (PRPP), the purine ring of ATP (the carbon from the formyl group of *N* ^10^-formyl-tetrahydrofolate (THF)), and glutamine (the nitrogen source). The 6-phosphogluconate, an intermediate of the ED/oxidative PP pathway, is the precursor of PRPP. In the oxidative PP pathway (Figure 4), the first (labeled) carbon in D-[1-^13^C]glucose is released as CO_2_ during the conversion of 6-phosphogluconate into D-ribulose-5-phosphate by 6-phosphogluconate dehydrogenase (PGD, EC 1.1.1.44), and it is unknown if the oxidative PP pathway is complete because the *pgd* gene in *R. denitrificans* is not annotated. Thus, the ^13^C-isotopomer abundance in histidine using D-[1-^13^C]glucose is possible from the following sources: (1) from the *N* ^10^-formyl moiety of THF if the oxidative PP pathway is active, (2) from D-ribose-5-phosphate or D-ribulose-5-phosphate derived from the non-oxidative PP pathway, where the labeled carbon is maintained (Figure 4), or both. The *N* ^10^-formyl moiety of THF is generated from THF and formate by transformylase, and various pathways can lead to formate biosynthesis in *R. denitrificans*. It is less likely that the labeled histidine using D-[1-^13^C]glucose was produced exclusively from the ^13^C-labeled formate through the oxidative PP pathway.

Alternatively, the non-oxidative PP pathways can also generate D-ribose-5-phosphate through steps of isomerization, epimerization and transketolation (Figure S3). If the metabolic flux goes through the non-oxidative PP pathway, more than one ^13^C-labeled sources are available using D-[6-^13^C]glucose (i.e. labeled GAP from the ED pathway and labeled fructose-6-phosphate (F6P)), while only F6P is labeled using D-[1-^13^C]glucose (GAP is not labeled via the ED pathway using D-[1-^13^C]glucose) (Figure 4). Since the EMP pathway in *R. denitrificans* is likely to be inactive, based on the experimental data and genomic information presented above, the labeled F6P is only led into the non-oxidative PP pathway. It is the most straightforward explanation for the detected ^13^C-labeling abundance in histidine (M+0 value is 0.28) with D-[1-^13^C]glucose (Table S1e) and for smaller M+0 value in histidine using D-[6-^13^C]glucose (0.10) (Table S1i) than using D-[1-^13^C]glucose (0.28). Together, our studies imply that *R. denitrificans* uses the non-oxidative PP pathway for histidine, ATP, coenzymes, and nucleic acids biosynthesis, as well as supplying GAP for the ED pathway (Figure 2a and 4).

### Carbon fixation in R. denitrificans

Previous reports suggested that *R. denitrificans* [19] and other AAPs [11], [20] can fix CO_2_. In our studies, no growth of *R. denitrificans* was observed in the minimal medium with CO_2_ (or bicarbonate) as the sole carbon source (Figure 1c), consistent with the finding that the key genes required for the Calvin cycle are missing in the genome of *R. denitrificans* OCh114 [8]. Moreover, no CO_2_-enhanced growth was obtained in the minimal medium containing pyruvate or glucose (Figure 1d and 1e), and ^13^C-isotopomer distribution of protein-based amino acids suggests that the presence of bicarbonate has a rather small effect on pyruvate and glucose metabolism in *R. denitrificans* (Table S1c and S1f). These experimental data indicate a carbon-fixation pathway other than the Calvin cycle is utilized by *R. denitirificans* OCh114. Swingley et al. [8] proposed that *R. denitrificans* could fix CO_2_ by pyruvate or/and PEP to form oxaloacetate (OAA). Using uniformly ^13^C-labeled D-glucose (D-[U-^13^C_6_] glucose) (Table S1j and S1k), our studies show less fully labeled amino acids derived from OAA (aspartate (M+4, 0.83), methionine (M+5, 0.78) and threonine (M+4, 0.85)) than the amino acid derived from pyruvate (alanine (M+3, 0.93)) (Table S1k). Also, a smaller M+0 value can be seen in threonine (0.86) and aspartate (0.87) than alanine (0.94) using ^13^C-NaHCO_3_ and unlabeled pyruvate (Table S1d). Similar results are also obtained using ^13^C-NaHCO_3_ and unlabeled glucose (Table S1g). Together, our studies imply that *R. denitrificans* fixes approximately 10–15% of protein carbon from CO_2_ via the anaplerotic pathways, despite lacking the Calvin cycle. These experimental evidences support the CO_2_ fixation pathways by *R. denitrificans* proposed by Swingley et al. [8].

### The TCA cycle and anaplerotic pathways

Our studies imply that *R. denitrificans* has an active TCA cycle and metabolic fluxes in the anaplerotic pathways. PEP can be converted into OAA, the precursor of aspartate, asparagine, methionine, threonine, lysine and isoleucine. OAA can be also generated from pyruvate and through the TCA cycle. Using D-[1-^13^C] glucose, the M+0 value is similar in aspartate (0.58), threonine (0.47), methionine (0.49) and alanine (0.56) but smaller than serine (0.74) (Table S1e), suggesting that ^13^C-isotopomer abundance in the amino acids derived from OAA is largely from pyruvate (via the anaplerotic pathway) and/or the TCA cycle flux.

Four anaplerotic enzymes are known to assimilate CO_2_ and replenish the intermediates of the TCA cycle, and genes encoding these anaplerotic enzymes can be all found in the genome of *R. denitrificans* OCh114; malic enzyme (*tme*, RD1_0421, EC 1.1.1.40, pyruvate
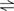
malate), PEP carboxykinase (*pckA*, RD1_1376, EC 4.1.1.49, PEP
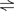
OAA), pyruvate carboxylase (*pyc*, RD1_3376, EC 6.4.1.1, pyruvate
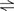
OAA) and PEP carboxylase (*ppc*, RD1_4248, EC 4.1.1.31, PEP
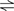
OAA). To further investigate carbon fixation by *R. denitrificans*, we used gene expression studies to confirm if the pathways are active and to identify the transcriptional level of the genes encoding enzymes for the pathways under various growth conditions, as well as ^13^C-isotopomer abundance to probe the anaplerotic flux. In the conditions we tested, the transcript level is higher for *pckA* (RD1_1376) and *tme* (RD1_0421) than for *pyc* (RD1_3376) and *ppc* (RD1_4248) (Figure 3). The activity of these four anaplerotic enzymes can be detected in cell-free crude extracts, consistent with the gene expression profiles. The activity is higher in PEP carboxykinase (6 umole/min·mg protein) and pyruvate carboxylase (2.5 umole/min·mg protein) than in PEP carboxylase (0.7 umole/ min·mg protein) in the cell extracts from the rich dark cultures, while the activity of pyruvate carboxylase and PEP carboxylase are comparable (1–2 umole/min·mg protein) in the minimal medium supplied with pyruvate.

**Figure 5:**
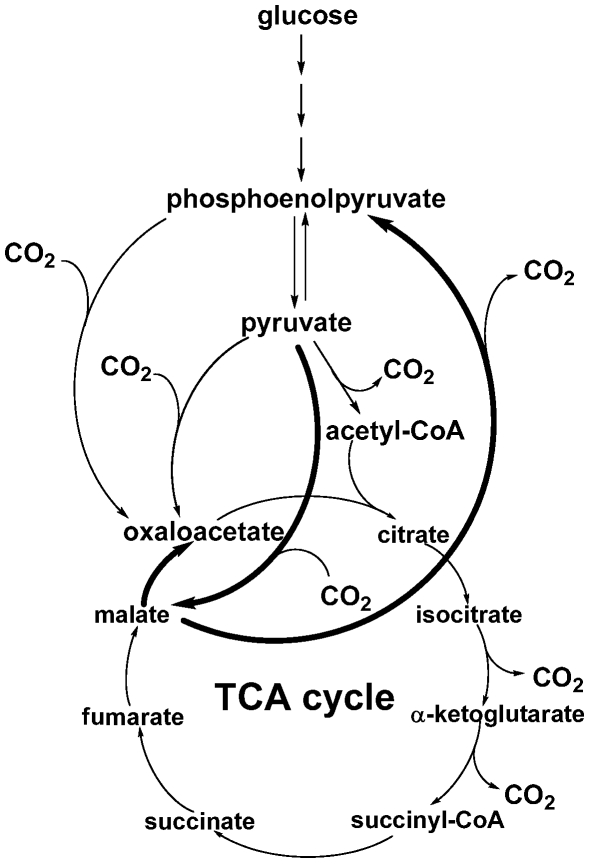
Proposed metabolic flux for carbon fixation by *R. denitrificans*. Bold curves represent proposed stronger metabolic fluxes.

To investigate metabolic fluxes through the anaplerotic pathways, [3-^13^C]pyruvate was used and the ^13^C-isotopomer abundance in aspartate (from OAA), serine (from PEP, 3-PGA) and alanine (from pyruvate) was analyzed (Table S1h). Both QRT-PCR and ^13^C-labeling pattern suggest that the flux of pyruvate to PEP is weak (different isotopomer distribution between alanine and serine), consistent with low gene expression of *ppdK* (RD1_1948, pyruvate phosphate dikinase, EC 2.7.9.1, pyruvate→PEP) and that the pathway from OAA to PEP is likely to be active and pyruvate to OAA is not as strong. This is in agreement with the gene expression profile of *pckA* (RD1_1376) and *pyc* (RD1_3376). Hence, most of the ^13^C-labeling in OAA likely comes from the TCA cycle flux (from malate), in which the pyruvate to malate pathway (via malic enzyme) should be rather active, compatible with higher expression of *tme* (RD1_0421) than *pyc* (RD1_3376). The proposed anaplerotic flux is summarized in Figure 5.

### The isoleucine and leucine biosynthesis pathways

**Figure 6:**
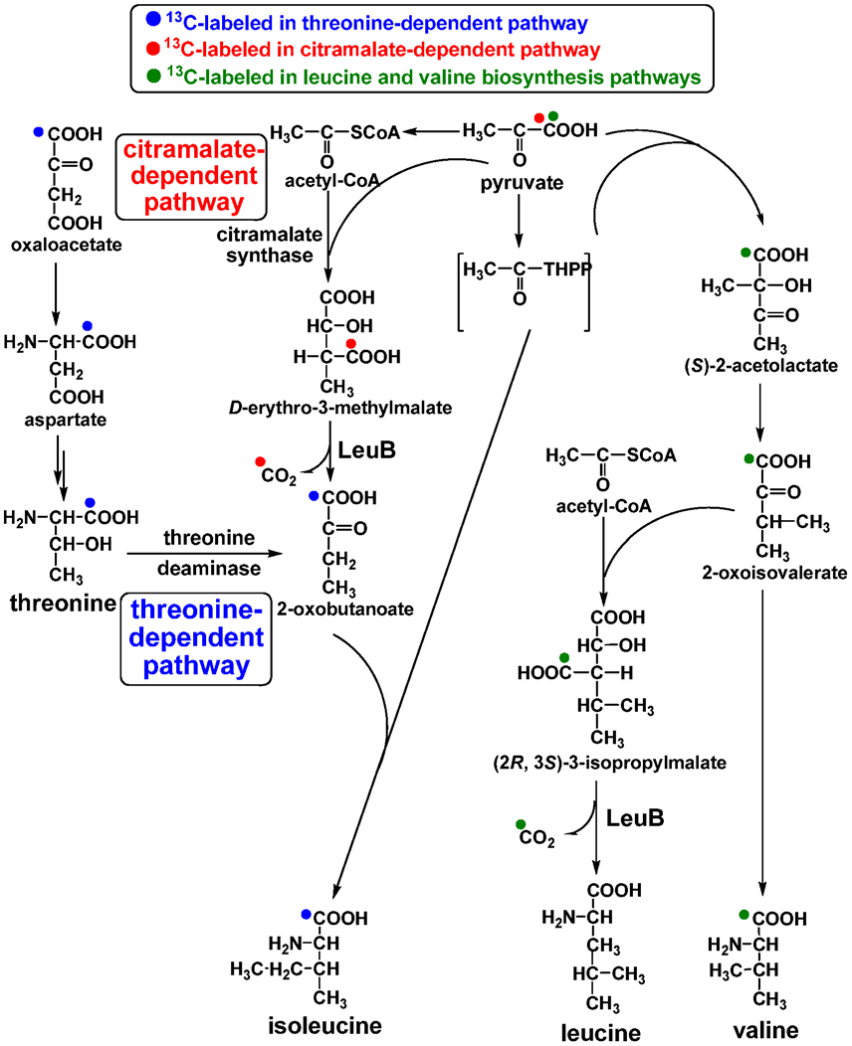
Proposed valine, leucine, and isoleucine biosynthesis pathways in *R. denitrificans*. Predicted ^13^C-labeling distributions using [1-^13^C]pyruvate in various pathways are shown. Experimentally identified ^13^C-labeling patterns are reported in the context.

In many organisms, isoleucine is converted from threonine by threonine deaminase (EC 4.3.1.19) in the threonine-dependent pathway [21], and leucine is made from valine. The ^13^C-isotopomer abundance in [1-^13^C]pyruvate cultures showed higher M+0 value in leucine (0.91) and isoleucine (0.86) compared to valine (0.25) and threonine (0.70) (Table S1a). Most of the leucine should be unlabeled because the first (labeled) carbon in ^13^C-(2*R*,3*S*)-3-isopropylmalate is released as CO_2_ catalyzed by 3-isopropylmalate dehydrogenase (LeuB, EC 1.1.1.85) (Figure 6). However, highly unlabeled isoleucine is not expected if 2-oxobutanoate, a precursor of isoleucine biosynthesis, is derived from threonine in the threonine-dependent pathway (Figure 6). The results suggest that isoleucine is not exclusively derived from threonine. If it is, the M+0 values in threonine and isoleucine should be similar.

Alternatively, a citramalate-dependent pathway, which is a threonine-independenet pathway, was recently reported in several bacteria [22]–[24]. These microorganisms can synthesize isoleucine from 2-oxobutanoate either exclusively from the citramalate-dependent pathway [24] or from both threonine- and citramalate-dependent pathways [23]. The higher M+0 value in isoleucine in our studies (Table S1a) can be explained if a significant portion of isoleucine is synthesized through the citramalate-dependent pathway, in which LeuB removes the first (labeled) carbon in ^13^C-D-*erythro*-3-methylmalate as CO_2_ (Figure 6). Similarly, the M+0 value is higher in isoleucine (0.90) than in threonine (0.77) using [1-^13^C]pyruvate and unlabeled NaHCO_3_ (Table S1c).

In the studies of the threonine-dependent pathway, QRT-PCR showed that the gene encoding theronine deaminase (*ilvA*, RD1_0416) was active in various growth conditions (Figure 3). The activity of citramalate synthase (CimA, EC 2.3.1.182) (3±0.5 nmole/min·mg protein) and theronine deaminase (60±5 nmole/min·mg protein) can be detected in the cell-free extracts of the minimal medium containing pyruvate. Further, smaller M+0 values in isoleucine (0.83–0.85) than in leucine (0.90–0.91) using [1-^13^C]pyruvate (Table S1a and S1b) imply that some isoleucine molecules are derived from the threonine rather than pyruvate and acetyl-CoA. If *R. denitrificans* utilizes the citramalate-dependent pathway exclusively to generate isoleucine, the ^13^C-labeling pattern in leucine and isoleucine will be identical (and the difference is expected to be within the error of GC-MS measurements: ∼0.01), since the same precursors (pyruvate and acetyl-CoA) are employed in the biosynthesis of both amino acids. Therefore, both threonine- and citramalate-dependent pathways should be contributed to synthesize isoleucine, considering that the difference in ^13^C-isotopomer abundance between leucine and isoleucine (0.06–0.07) is larger than the instrumental error (∼0.01). Further, the calculated values of all ^13^C-isotopomer abundance (from M+0 to M+5) of isoleucine indicate that 80% of the *in vivo* metabolic flux for isoleucine biosynthesis comes from the citramalte-dependent pathway, 20% from the threonine-dependent pathway, when [3-^13^C]pyruvate is the carbon source (Table S1h and Figure S3). Together, our studies suggest that both the citramalate- and threonine-dependent pathways are active for isoleucine biosynthesis in *R. denitrificans*, similar to the report in *Geobacter sulfurreducens* [23]. As the putative *cimA* gene is not annotated in *R. denitrificans*, identification of the proposed citramalate synthase is in progress.

## Discussion

### CO_2_ fixation in R. denitrificans and other AAPs

In this paper, we reported that *R. denitrificans* uses the anaplerotic pathways to fix 10–15% of protein carbon from CO_2_. The amount of CO_2_ fixation is lower in *R. denitrificans* than in autotrophic bacteria, supporting previous observations that *R. denitrificans* cannot grow autotrophically. Other than *R. denitrificans*, CO_2_ fixation in several AAPs producing BChl *a* aerobically was reported previously by ^14^CO_2_ incorporation studies: *Erythrobacter sibiricum* (a very low level of CO_2_ (0.4%) assimilated) [7], *Erythrobacter longus* [7], *Acidiphilium rubrum* [20] and *Erythrobacter* sp. strains [15]. Some difference in CO_2_ fixation can be detected between *R. denitrificans* and *A. rubrum*. In *A. rubrum*, CO_2_ fixation is completely inhibited with the glucose-growth culture, and is enhanced 3- to 5-fold in the light (∼3.0 nmoles/mg dry cell weight) [20]. In contrast, our studies suggest that CO_2_ incorporation can be detected in *R. denitrificans* with either pyruvate- or glucose-growth cultures in either light or dark growth, and is not enhanced by light exposure.

Among the wide-spread marine *Roseobacter* clade, many of them cannot generate photosynthetic pigments. To the best of our knowledge, no CO_2_ assimilation data have been reported for these unpigmented strains of the *Roseobacter* clade. It will be very useful to compare the difference in the CO_2_ assimilation between *R. denitrificans* and un-pigmented *Roseobacter* clade bacteria.

### Carbohydrate metabolism in R. denitrificans

The ED pathway has generally been viewed as an alternative to the EMP pathway. In examining the carbohydrate catabolism of both *Rhodobacter capsulatus* [25] and *Rhodobacter sphaeroides* [26] under anaerobically phototrophic and aerobically heterotrophic growth conditions, Conrad and Schlegel reported that fructose degradation was shifted to the ED pathway from the EMP pathway during the transition from anaerobically phototrophic to aerobically heterotrophic growth conditions, and that glucose catabolism was going through the ED pathway under both phototrophic and heterotrophic growth. In agreement with their experimental evidences, all of the genes in the EMP and ED pathways can be found in the genome of several *R. sphaeroides* strains, while the 6-phosphogluconate dehydrogenase gene (*pgd*) in the oxidative PP pathway cannot be annotated.

Our studies in this report indicate that the ED pathway plays a significant role in glucose metabolism of *R. denitrificans*, and that the non-oxidative PP pathway contributes to histidine, ATP, coenzyme and nucleic acid biosynthesis, and that no metabolic flux going through the EMP pathway and the oxidative PP pathway. Our experimental evidences are consistent with the genomic information of *R. denitrificans*, in which the ED pathway and the non-oxidative PP pathway are complete, but the *pfk* gene, essential for the EMP pathway, and the *pgd* gene, required for the oxidative PP pathway, are not annotated.

Searching the genomes of the *Roseobacter* clade [5], [8]–[10], all of them have genes in the ED pathway annotated. The ED pathway is identified mainly in prokaryotes, and almost all of them are aerobes and facultative bacteria, consistent with the physiological features of AAPs. Moreover, note that the *pgd* gene is also missing in *Silicibacter pomeroyi* DSS-3, *Silicibacter* sp. TM1040 and *Dinoroseobacter shibae* DFL12, and that the *pfk* gene is not found in *Jannaschia* sp. CCS1, *Silicibacter pomeroyi* DSS-3, and *Silicibacter* sp. TM1040. Alternatively, *Dinoroseobacter shibae* DFL12 has all of the genes in the EMP pathway, and *Jannaschia* sp. CCS1 has complete oxidative PP pathway. It will be interesting to learn carbohydrate metabolisms in these two bacteria.

### Possible physiological roles of the ED pathway in R. denitrificans

The physiological significance for *R. denitrificans* and possibly some other resourceful heterotrophic marine *Roseobacter* clade [12] to depend on the ED pathway, which produces 1 ATP, 1 NADH and 1 NADPH, compared to 2 ATP and 2 NADH from the more common EMP pathway, is an interesting question. The most straightforward answer could be that *R. denitrificans* demands more NADPH than ATP in the cells when the oxidative PP pathway is not active. The oxidative PP pathway, not the non-oxidative PP pathway, can generate two molecules of NADPH from one molecule of glucose, and can produce approximately 60% of the NADPH required inside the cells.

NADPH is not only the reducing power for synthesizing energy-rich molecules and biofuels, but also prevents oxidative stress by reducing glutathione via glutathione reductase, which converts reactive H_2_O_2_ into H_2_O by glutathione peroxidase [27]. The reactive oxygen species inside the cell can be fatal for *R. denitrificans* since it is an aerobic anoxygenic bacterium that contains highly absorbing pigments that can sensitize reactive oxygen species formation. When the oxidative PP pathway is not active, the ED pathway may be the best option to generate NADPH required by the cells. It was not established whether an anoxygenic photosynthetic bacterium, like *R. denitrificans*, can produce glutathione, which can be synthesized by oxygenic phototrophs, such as cyanobacteria and higher plants. Note that genes for glutathione biosynthesis (glutamate-cysteine ligase, *gshA*, RD1_4077, glutathione synthetase, *gshB*, RD1_1192), glutathione reductase (*gor*, RD1_1919), glutathione peroxidase (*gpo*, RD1_0599), and some glutathione transferases can be found in *R. denitrificans* genome, suggesting possible roles of glutathione in reducing oxidative stress in *R. denitrificans*.

The other possibility for *R. denitrificans* adapting the ED pathway could be that many marine *Roseobacter*-lineage bacteria live in coastal seawater, an environment enriched with a wider variety of organic acids, and the ED pathway is a better option for digesting the hexuronic acids (e.g. glucuronic acid). This hypothesis may be supported by the broad substrate specificity of KDPG aldolase identified biochemically [28] and structurally [29]. As gene sequencing is currently in progress for 40 different *Roseobacter* strains [2], it will be interesting to learn if the patterns of carbohydrate metabolism revealed in this work may be a general theme in the *Roseobacter* clade, especially the significance of the ED/non-oxidative PP pathway in these physiologically diverse, widespread and high abundance marine microorganisms.

### Gene expression under different growth conditions

Throughout the QRT-PCR data analysis, we observed that the transcript level for all of the genes tested is higher in cells grown in the minimal medium containing either pyruvate or glucose than in the rich medium (Figure 3). The discrepancies may be attributed to the following possible scenarios: (1) metabolic regulation: it has been thought in some bacteria, *E. coli* included, that the anaplerotic and metabolic pathways are not as much in demand in the rich medium as in the minimal medium containing defined carbon source, or some pathways may be shut down due to metabolic regulation [30], [31], so genes in those metabolic pathways are not highly expressed in rich growth medium; and/or (2) the abundance of the 16S rRNA gene in different cell growth conditions: as reported in previous studies [32]–[34], the abundance of the 16S rRNA gene is dependent on the growth rate of the cells. If it is the case for *R. denitrificans*, lower copy numbers of the 16S rRNA gene will be expected in the minimal medium with glucose (grow slowest), and highest number in the rich medium grown under dark conditions (grow fastest). Together, both possible scenarios can explain lower transcript level for all of the target genes we tested in the rich medium. Further investigations are required for addressing this important issue.

### Requirement of yeast extract or exogenous vitamin B_12_

In this report, we demonstrate that vitamin B_12_ can successfully replace the undefined carbon source, yeast extract, for the growth of *R. denitrificans* in our developed minimal medium containing defined organic carbon source. While it is not known why the undefined carbon source yeast extract is required for *R. denitrificans* and several other photosynthetic bacteria, it is not clear why exogenous vitamin B_12_ is required for *R. denitrificans*, because all of the genes required for vitamin B_12_ biosynthesis are annotated in *R. denitrificans* (based on the information in the KEGG (Kyoto Encyclopedia of Genes and Genomes) database). It is also unclear how cobalamin can be utilized by *R. denitrificans*, since no known cobalamin transporter genes (*btuB*, *btuC*, *btuD* and *btuF*) can be recognized in the genome. Nonetheless, the effect of exogenous vitamin B_12_ is clear and its inclusion permitted most of the results reported in this paper. More experimental evidences are required for understanding the roles of yeast extract and exogenous vitamin B_12_ reported herein for enhancing cell growth.

### A common feature in Roseobacter clade?

Our studies suggest approximately 10–15% CO_2_ fixation in protein carbon through the anaplerotic enzymes by *R. denitrificans*, supporting the proposal by Swingley et al. [8]. We also confirmed that unlike carbon fixation by autotrophs, *R. denitrificans* cannot live with CO_2_ as the sole carbon source, consistent with lack of genes for key enzymes of the Calvin cycle. Missing genes in the Calvin cycle are also common features in the other four *Roseobacter* clade bacteria with complete sequences [2], [5], [9], suggesting that carbon fixation mechanisms other than the Calvin cycle are needed for the AAPs.

Other than the ED pathway, our studies demonstrate that *R. denitrificans* uses the non-oxidative PP pathway as the other alternative pathway for sugar degradation to make the ribose-5-phosphate for histidine, cofactors, ATP and nucleic acid biosynthesis, as well as to produce GAP for carbon metabolism. In contrast to the oxidative PP pathway, no CO_2_ is released in the non-oxidative PP pathway, which is an important feature for carbon fixation by autotrophs using the Calvin cycle. In these autotrophs, the non-oxidative PP pathway is part of the Calvin cycle. Although *R. denitrificans* cannot use the Calvin cycle for fixing CO_2_, it does have a complete non-oxidative PP pathway and relies on the pathway for converting hexose phosphate into pentose phosphate.

## Materials and Methods

### Materials

The DNA oligomers in this report are from Integrated DNA Technology (IDT) and were used without further purification. The ^13^C-labeled glucose, pyruvate and sodium bicarbonate were purchased from Cambridge Isotope Laboratories (CIL), Inc (MA, USA).

### The growth of bacterial strains

*R. denitrificans* OCh114 (a gift from the laboratory of Dr. Beatty at University of British Columbia, Vancouver, Canada) was grown aerobically (20–30%-filled Erlenmeyer flasks) on either a nutrition-rich medium (Difco™ Marine Broth 2216; Becton, Dickson and Company) (pH 7.5) or a reported minimal medium [13] with modifications (illustrated in next paragraph). No undefined carbon sources (yeast extract) was included in our minimal growth medium. The seawater source in our minimal medium was prepared by 3.2% Instant Ocean® artificial sea salts (United Pet Group, Cincinnati, OH). The minimal growth medium was adjusted to pH 7.5. All of the cultures were grown at 28°C and the growth rates were estimated by OD_600_. We used 2% cultures (50-fold dilution) in the late exponential growth phase grown in the rich medium to inoculate the minimal medium containing defined carbon source(s). To minimize the carryover of the rich medium, inoculated cells were centrifuged and supernatant liquid was removed. Cell pellets were washed with the minimal medium (no carbon source included) twice, resuspended in the minimal medium, and then transferred to the minimal medium containing defined carbon source(s).

In the minimal medium supplied with defined ^13^C-isotopically labeled carbon source, 0.1% pyruvate (labeled on either the first or third carbon position), 0.1% glucose (on the first, sixth or uniform labeled), or 0.2% NaH^13^CO_3_ were added. Due to the requirement of a growth medium without including undefined carbon sources for GC/MS studies, ferric citrate [13] was replaced by ferric chloride hexahydrate, and yeast extract was substituted by 20 µg/L vitamin B_12_. The cultures were grown to an exponential phase, representing the pseudo steady-state, and then harvested for analyzing the isotopomer distribution in key metabolites.

### RNA extraction and quantitative real-time PCR (QRT-PCR)

The methods used to extract RNA and perform QRT-PCR were described previously [35]. QRT-PCR was performed to profile the gene expression under different growth conditions of *R. denitrificans* OCh114. cDNA was synthesized from 1 µg RNA and 100 µM random 9mer DNA using Superscript III reverse transcriptase (Invitrogen). The QRT-PCR reactions were performed via ABI 7500 Real-Time PCR System (Applied Biosystems). The primers (shown in Table S2) for QRT-PCR reactions were designed using Primer Express 2.0 software (PE Applied Biosystems) and analyzed by OligoAnalyzer 3.0 software (IDT). The Power SYBR Green Master Mix (PE Applied Biosystem) was used for amplifying DNA [35]. The cycle threshold (Ct) was calculated as the cycle number at which a fluorescence threshold (ΔRn) crossed the baseline. Data were normalized by analyzing ΔCt = Ct of the target gene – Ct of the internal control gene (16S rRNA), and each relative gene expression value is calculated with 2^−(ΔCt)^. Three biological replicates, with six technical replicates for each biological sample, and total eighteen technical replicates were performed for validation, and the mean value was reported (Figure 3). The amplified DNA fragments were verified by 1% agarose gel electrophoresis, and a single fragment was obtained for all amplicons (data not shown).

### Measurements of pyruvate/glucose uptake

The amount of D-glucose in solution was determined by hexokinase/glucose-6-phosphate dehydrogenase coupling assay with a D-glucose assay kit (Roche, Mannheim, Germany), and the reduction of NAD^+^ was followed spectrophotometrically by the increase at 340 nm in absorbance. The amount of pyruvate in the culture was estimated by lactate dehydrogenase (EC 1.1.2.3) (Sigma-Aldrich) with 25 µM NADH in 0.1 M Tris-HCl buffer at pH 8.0 [36]. The reaction was followed by the oxidation of NADH (the decrease at 340 nm in absorbance).

### Enzyme assays

Enzymatic activities were performed with cell-free crude extracts prepared as follows. Cells were harvested by centrifugation at 5,000×*g* for 15 min at 4°C and washed with 20 mM Tris-HCl buffer at pH 8.0. Cell pellet was resuspended in the same buffer containing 1 mM phenylmethanesulfonyl fluoride (PMSF). Resuspended cells were disrupted by sonication, and cell debris was removed with centrifugation at 20,000×*g* for 30 min. Protein concentration in cell extracts was determined by the Bradford assay [37] using bovine serum albumin as a standard. The activity of pyruvate carboxylase, phosphoenolpyruvate carboxylase, phosphoenolpyruvate carboxykinase, malic enzyme, citramalate synthase, 6-phosphofructokinase, 2-keto-3-deoxy-6-phosphogluconate aldolase, 6-phosphogluconate dehydrase, isopropylmalate synthase and threonine deaminase in cell extracts were assayed as described previously [22], [23], [38]–[46].

### Gas chromatography (GC)/mass spectrometry (MS)

The methods for GC/MS are reported previously [44], [47]–[49]. In brief, biomass was collected from 50 mL culture by centrifuge and then was hydrolyzed in 6 M HCl at 100°C. The resulting amino acids were dried and derivatized in tetrahydrofuran and N-(*tert*-butyl dimethylsilyl)-N-methyl-trifluoroacetamide (Sigma-Aldrich) at 70°C for 1 h. A gas chromatograph (GC) (Hewlett-Packard, model 7890A, Agilent Technologies Inc., Ballwin, MO) equipped with a DB5-MS column (J&W scientific, Folsom, CA) and a mass spectrometer (5975C, Agilent Technologies Inc.) was used for analyzing metabolite labeling profiles. We report four types of MS fragment data: (1) the [M-15]^+^ group, which loses a methyl group; (2) the [M-57]^+^ group, containing un-fragmented amino acids; (3) the [M-159]^+^ group, which contains amino acids losing α carboxyl group; and (4) the [M-302]^+^ group, containing only the 1^st^ (α carboxyl group) and 2^nd^ carbons in the amino acid backbone (Note that [M-302]^+^ and [M-15]^+^ cannot be detected in some amino acids). Three labeling data were used to trace the carbon metabolic route and to identify active pathways. Throughout the article, M+0, M+1, M+2… stands for the value of unlabeled (M+0) and of additional ^13^C-labeled protein-derived amino acids (M+1, M+2….).

## Supporting Information

Figure S1 Spectra, culture, and growth curves of R. denitrificans under different growth conditions. The culture of R. denitrificans grown in the rich medium under the day-light cycles (a). The spectrum (b) and growth curve (c) of the cells grown in the rich medium under dark versus under light. The spectrum of the cells grown under dark and day-light cycle is shown in the inset. The formation of the light harvesting antenna complex II antenna complex (807-nm peak) is repressed in the light, while the level of the reaction center-light harvesting complex I (RC-LHI) core complex (872-nm peak) is similar. Data fit of pyruvate uptake with or without NaHCO3 in the defined medium, and the uptake rate is 2.5×10−2±5×10−4 mmole per hour (d).
(1.12 MB TIF)

Figure S2 The non-oxidative pentose phosphate pathway. All of the reaction steps are reversible. Possible 13C-labeling using [1-13C]glucose or [6-13C]glucose is shown in green or red dots, respectively.
(0.47 MB TIF)

Figure S3 Experimental values of the isotopomer abundance of isoleucine (red bar) using [3-13C]pyruvate as the defined carbon source, and predicted contributions of the threonine (back bar) and citramalate (blue bar) pathways for isoleucine biosynthesis in R. denitrificans.
(0.62 MB TIF)

Table S1 13C-isotopomer abundances of tert-butyl dimethylsilyl (TBDMS) derivatives of protein-derived amino acids from R. denitrificans OCh114 grown in the defined medium containing different carbon sources. Except in (b), all of the cultures were grown in the dark. The unlabeled molecule is shown as M+0, and additional mass comes from 13C-labeled carbon source. All of the data, except leucine and isoleucine, are [M-57]+ data (tert-butyl group is removed). The [M-15]+ data (methyl group is cleaved) for leucine and isoleucine are shown, because the [M-57]+ data are overlapped in GC-MS.
(0.27 MB DOC)

Table S2 Primers used for QRT-PCR studies reported in this paper
(0.04 MB DOC)

Table S3 The theoretical and experimental M+0 values in alanine and serine using D-[1-13C] glucose and D-[6-13C]glucose^a,b^
(0.03 MB DOC)
