# Supplementary figures and images for: Carbohydrate Metabolism and Carbon Fixation in Roseobacter denitrificans OCh114
Source: PLoS One. 2009 Oct 1;4(10):e7233. doi: 10.1371/journal.pone.0007233 (PMC2749216; doi:10.1371/journal.pone.0007233)

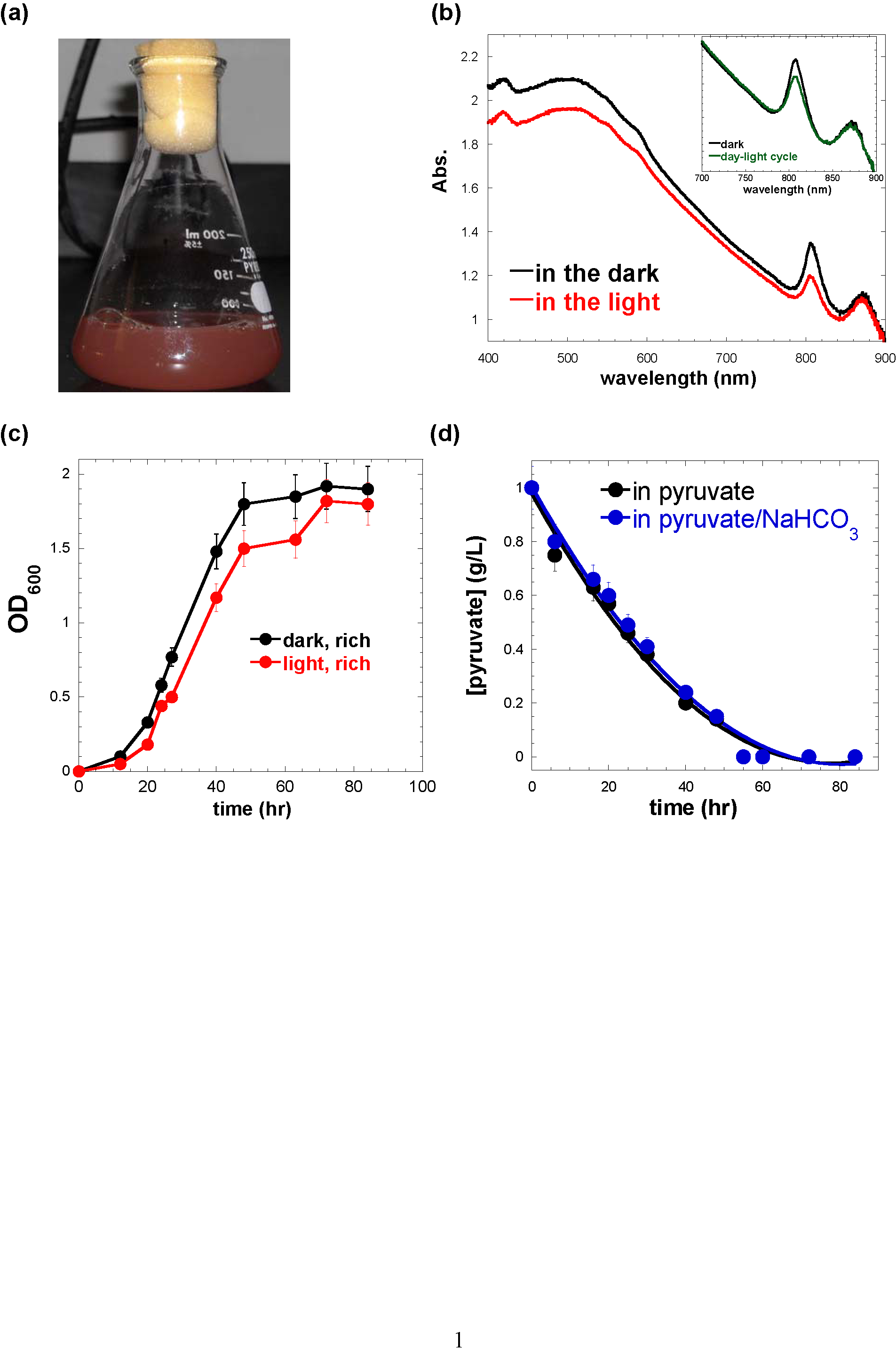

Supplement: Figure S1 — Spectra, culture, and growth curves of R. denitrificans under different growth conditions. The culture of R. denitrificans grown in the rich medium under the day-light cycles (a). The spectrum (b) and growth curve (c) of the cells grown in the rich medium under dark versus under light. The spectrum of the cells grown under dark and day-light cycle is shown in the inset. The formation of the light harvesting antenna complex II antenna complex (807-nm peak) is repressed in the light, while the level of the reaction center-light harvesting complex I (RC-LHI) core complex (872-nm peak) is similar. Data fit of pyruvate uptake with or without NaHCO3 in the defined medium, and the uptake rate is 2.5×10^−2^±5×10^−4^ mmole per hour (d). (1.12 MB TIF) [file pone.0007233.s001.tif]

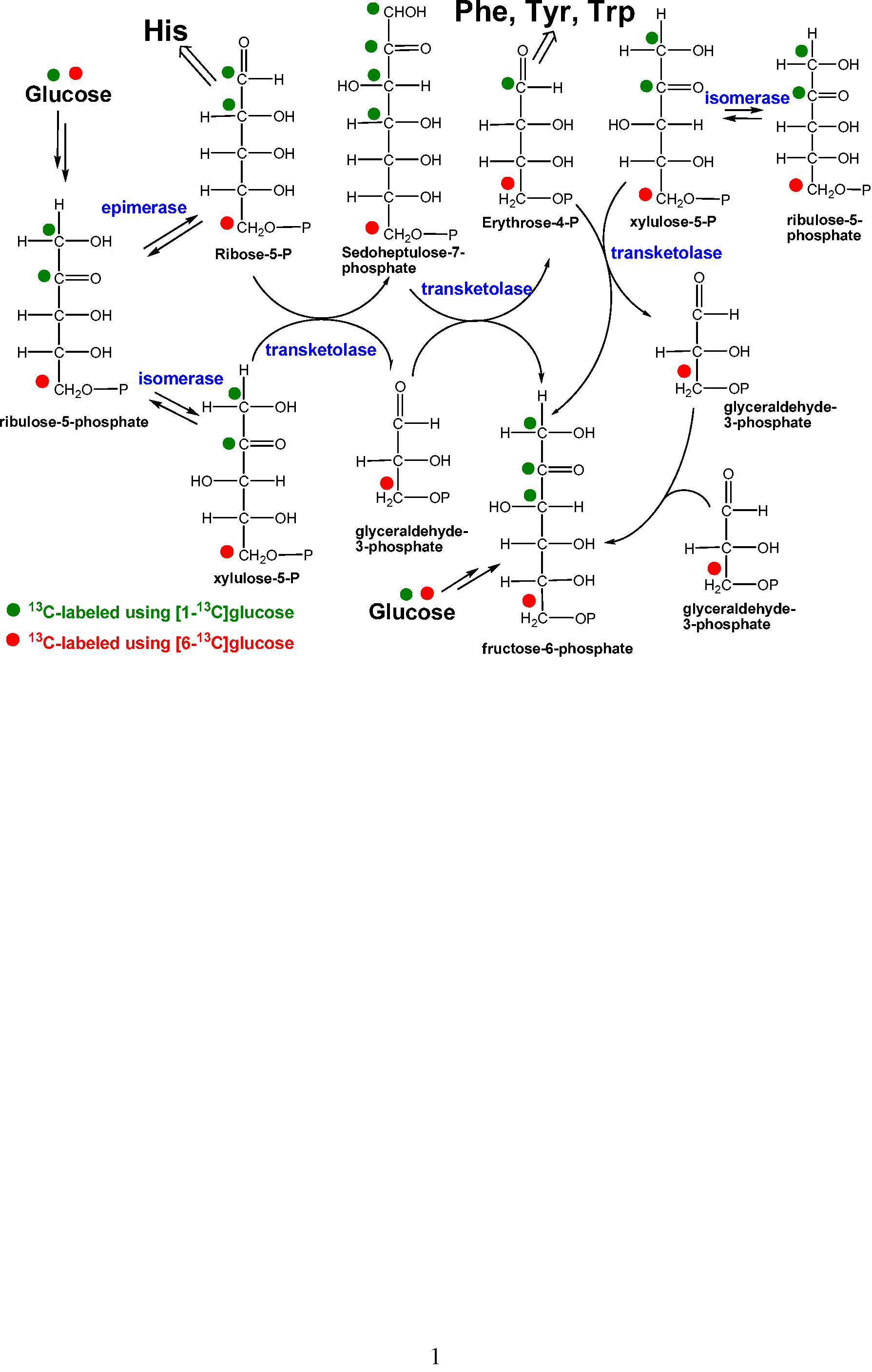

Supplement: Figure S2 — The non-oxidative pentose phosphate pathway. All of the reaction steps are reversible. Possible 13C-labeling using [1-13C]glucose or [6-13C]glucose is shown in green or red dots, respectively. (0.47 MB TIF) [file pone.0007233.s002.tif]

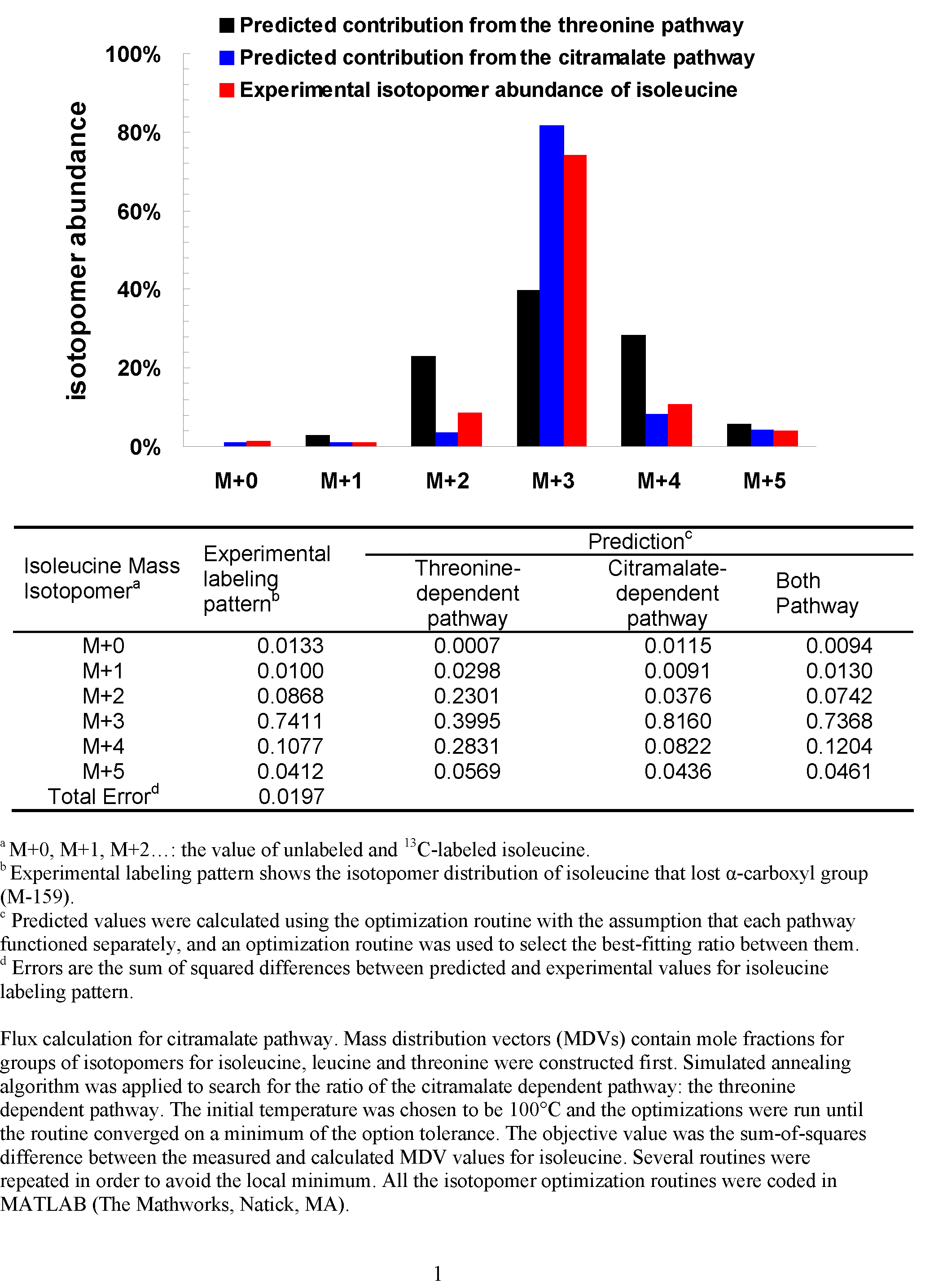

Supplement: Figure S3 — Experimental values of the isotopomer abundance of isoleucine (red bar) using [3-13C]pyruvate as the defined carbon source, and predicted contributions of the threonine (back bar) and citramalate (blue bar) pathways for isoleucine biosynthesis in R. denitrificans. (0.62 MB TIF) [file pone.0007233.s003.tif]
