# Supplementary material for: Carbohydrate Metabolism and Carbon Fixation in Roseobacter denitrificans OCh114
Source: PLoS One. 2009 Oct 1;4(10):e7233. doi: 10.1371/journal.pone.0007233 (PMC2749216; doi:10.1371/journal.pone.0007233)
Supplement: Table S1 — 13C-isotopomer abundances of tert-butyl dimethylsilyl (TBDMS) derivatives of protein-derived amino acids from R. denitrificans OCh114 grown in the defined medium containing different carbon sources. Except in (b), all of the cultures were grown in the dark. The unlabeled molecule is shown as M+0, and additional mass comes from 13C-labeled carbon source. All of the data, except leucine and isoleucine, are [M-57]+ data (tert-butyl group is removed). The [M-15]+ data (methyl group is cleaved) for leucine and isoleucine are shown, because the [M-57]+ data are overlapped in GC-MS. (0.27 MB DOC) [file pone.0007233.s004.doc]

**(a)** 0.1% [1-^13^C]pyruvate, dark

|  | **Ala** | **Gly** | **Val** | **Leu** | **Ile** | **Ser** | **Met** | **Thr** | **Asp** | **Glu** | **His** | **Phe** | **Tyr** |
| --- | --- | --- | --- | --- | --- | --- | --- | --- | --- | --- | --- | --- | --- |
| **M+0** | 0.2787 | 0.7838 | 0.2475 | 0.9146 | 0.8518 | 0.7598 | 0.6679 | 0.7014 | 0.7241 | 0.8398 | 0.6804 | 0.5188 | 0.5166 |
| **M+1** | 0.7185 | 0.2178 | 0.7289 | 0.0700 | 0.1008 | 0.2322 | 0.2203 | 0.1846 | 0.1757 | 0.1577 | 0.2899 | 0.3119 | 0.3131 |
| **M+2** | 0.0053 | 0 | 0.0247 | 0.0009 | 0.0327 | 0.0092 | 0.1073 | 0.1152 | 0.1014 | 0.001 | 0.0218 | 0.1399 | 0.156 |
| **M+3** | 0 |  | 0 | 0.0143 | 0.0034 | 0 | 0.005 | 0.0047 | 0 | 0.0017 | 0.0071 | 0.0292 | 0.0044 |
| **M+4** |  |  | 0.0008 | 0.0050 | 0 |  | 0.0053 | 0 | 0 | 0.0001 | 0.003 | 0.0002 | 0.012 |
| **M+5** |  |  | 0.0003 | 0.0029 | 0.0005 |  | 0 |  |  | 0 | 0 | 0 | 0.0012 |
| **M+6** |  |  |  | 0 | 0.0004 |  |  |  |  |  | 0 | 0.0007 | 0 |
| **M+7** |  |  |  |  |  |  |  |  |  |  |  | 0 | 0.0004 |
| **M+8** |  |  |  |  |  |  |  |  |  |  |  | 0 | 0.0003 |
| **M+9** |  |  |  |  |  |  |  |  |  |  |  | 0 | 0 |

**(b)** 0.1% [1-^13^C]pyruvate, light

|  | **Ala** | **Gly** | **Val** | **Leu** | **Ile** | **Ser** | **Met** | **Thr** | **Asp** | **Glu** | **His** | **Phe** | **Tyr** |
| --- | --- | --- | --- | --- | --- | --- | --- | --- | --- | --- | --- | --- | --- |
| **M+0** | 0.2891 | 0.8038 | 0.2604 | 0.8997 | 0.8295 | 0.8032 | 0.711 | 0.7448 | 0.7616 | 0.8445 | 0.7204 | 0.547 | 0.5421 |
| **M+1** | 0.6954 | 0.1953 | 0.7073 | 0.0559 | 0.1342 | 0.2 | 0.1964 | 0.1719 | 0.169 | 0.1481 | 0.2527 | 0.3333 | 0.3314 |
| **M+2** | 0.0146 | 0.0008 | 0.033 | 0.0108 | 0.0376 | 0 | 0.1846 | 0.0816 | 0.0667 | 0.0076 | 0.0286 | 0.1051 | 0.1152 |
| **M+3** | 0.0009 |  | 0 | 0.0326 | 0.0068 | 0 | 0.0059 | 0 | 0.0023 | 0 | 0 | 0.0141 | 0.0101 |
| **M+4** |  |  | 0 | 0.0060 | 0 |  | 0.0009 | 0.0036 | 0.0003 | 0 | 0 | 0.0001 | 0 |
| **M+5** |  |  | 0 | 0 | 0.0004 |  | 0.0012 |  |  | 0.0002 | 0 | 0.0009 | 0.001 |
| **M+6** |  |  |  | 0.0001 | 0.0005 |  |  |  |  |  | 0 | 0 | 0.0008 |
| **M+7** |  |  |  |  |  |  |  |  |  |  |  | 0 | 0 |
| **M+8** |  |  |  |  |  |  |  |  |  |  |  | 0 | 0 |
| **M+9** |  |  |  |  |  |  |  |  |  |  |  | 0 | 0 |

**(c)** 0.1% [1-^13^C]pyruvate and 0.2% NaHCO_3_

|  | **Ala** | **Gly** | **Val** | **Leu** | **Ile** | **Ser** | **Met** | **Thr** | **Asp** | **Glu** | **His** | **Phe** | **Tyr** |
| --- | --- | --- | --- | --- | --- | --- | --- | --- | --- | --- | --- | --- | --- |
| **M+0** | 0.2542 | 0.8021 | 0.073 | 0.9364 | 0.9042 | 0.7971 | 0.7471 | 0.7713 | 0.7903 | 0.8972 | 0.7348 | 0.5095 | 0.5378 |
| **M+1** | 0.7334 | 0.1972 | 0.8895 | 0.0484 | 0.0843 | 0.2038 | 0.1993 | 0.1807 | 0.1651 | 0.1022 | 0.2354 | 0.3576 | 0.3537 |
| **M+2** | 0.0128 | 0.0007 | 0.0426 | 0.0215 | 0.0135 | 0 | 0.0558 | 0.038 | 0.0412 | 0 | 0.0333 | 0.1155 | 0.0967 |
| **M+3** | 0 |  | 0 | 0 | 0.0026 | 0 | 0 | 0.01 | 0.002 | 0 | 0 | 0.0198 | 0.0121 |
| **M+4** |  |  | 0 | 0 | 0 |  | 0 | 0 | 0.0014 | 0 | 0 | 0 | 0 |
| **M+5** |  |  | 0 | 0.0006 | 0.0004 |  | 0 |  |  | 0 | 0 | 0 | 0 |
| **M+6** |  |  |  | 0.0002 | 0.0003 |  |  |  |  |  | 0 | 0 | 0 |
| **M+7** |  |  |  |  |  |  |  |  |  |  |  | 0 | 0 |
| **M+8** |  |  |  |  |  |  |  |  |  |  |  | 0 | 0 |
| **M+9** |  |  |  |  |  |  |  |  |  |  |  | 0 | 0 |

**(d)** 0.2% ^13^C-NaHCO_3_ and 0.1% unlabeled pyruvate

|  | **Ala** | **Gly** | **Val** | **Leu** | **Ile** | **Ser** | **Met** | **Thr** | **Asp** | **Glu** | **His** | **Phe** | **Tyr** |
| --- | --- | --- | --- | --- | --- | --- | --- | --- | --- | --- | --- | --- | --- |
| **M+0** | 0.9427 | 0.9473 | 0.951 | 0.8878 | 1.000 | 0.9189 | 0.9149 | 0.8633 | 0.8683 | 0.8864 | 0.9517 | 0.854 | 0.8383 |
| **M+1** | 0.05 | 0.0554 | 0.0478 | 0.0638 | 0 | 0.0762 | 0.0742 | 0.1379 | 0.1328 | 0.1186 | 0.0023 | 0.1382 | 0.1194 |
| **M+2** | 0.005 | 0 | 0.0074 | 0.0363 | 0 | 0.0029 | 0.0225 | 0 | 0 | 0 | 0.109 | 0.0111 | 0.06 |
| **M+3** | 0.0024 |  | 0 | 0.0237 | 0 | 0.002 | 0 | 0 | 0 | 0 | 0 | 0.0006 | 0 |
| **M+4** |  |  | 0 | 0 | 0 |  | 0 | 0 | 0 | 0 | 0 | 0 | 0 |
| **M+5** |  |  | 0 | 0 | 0 |  | 0 |  |  | 0 | 0 | 0.0003 | 0 |
| **M+6** |  |  |  | 0.008 | 0 |  |  |  |  |  | 0 | 0.0003 | 0 |
| **M+7** |  |  |  |  |  |  |  |  |  |  |  | 0 | 0 |
| **M+8** |  |  |  |  |  |  |  |  |  |  |  | 0 | 0 |
| **M+9** |  |  |  |  |  |  |  |  |  |  |  | 0 | 0 |

**(e)** 0.1% D-[1-^13^C]glucose

|  | **Ala** | **Gly** | **Val** | **Leu** | **Ile** | **Ser** | **Met** | **Thr** | **Asp** | **Glu** | **His** | **Phe** | **Tyr** |
| --- | --- | --- | --- | --- | --- | --- | --- | --- | --- | --- | --- | --- | --- |
| **M+0** | 0.5629 | 0.8943 | 0.4146 | 0.4191 | 0.4677 | 0.7369 | 0.4889 | 0.4666 | 0.5764 | 0.3947 | 0.2788 | 0.4793 | 0.5286 |
| **M+1** | 0.3994 | 0.0983 | 0.4438 | 0.3457 | 0.3002 | 0.2437 | 0.2883 | 0.3756 | 0.3139 | 0.3754 | 0.5393 | 0.3197 | 0.2929 |
| **M+2** | 0.0365 | 0.0074 | 0.1305 | 0.1949 | 0.1813 | 0.02 | 0.1674 | 0.1448 | 0.0978 | 0.1858 | 0.1464 | 0.1524 | 0.1472 |
| **M+3** | 0.0012 |  | 0.0086 | 0.0329 | 0.0521 | 0 | 0.0276 | 0.0128 | 0.012 | 0.0395 | 0.0347 | 0.039 | 0.0382 |
| **M+4** |  |  | 0.0026 | 0.0074 | 0.0087 |  | 0.0145 | 0.0003 | 0 | 0.005 | 0 | 0.0083 | 0.0017 |
| **M+5** |  |  | 0 | 0.0022 | 0 |  | 0.0134 |  |  | 0 | 0 | 0.0015 | 0.0007 |
| **M+6** |  |  |  | 0 | 0.0003 |  |  |  |  |  | 0 | 0 | 0.0007 |
| **M+7** |  |  |  |  |  |  |  |  |  |  |  | 0 | 0 |
| **M+8** |  |  |  |  |  |  |  |  |  |  |  | 0 | 0 |
| **M+9** |  |  |  |  |  |  |  |  |  |  |  | 0 | 0 |

**(f)** 0.1% D-[1-^13^C]glucose and 0.2% NaHCO_3_

|  | **Ala** | **Gly** | **Val** | **Leu** | **Ile** | **Ser** | **Met** | **Thr** | **Asp** | **Glu** | **His** | **Phe** | **Tyr** |
| --- | --- | --- | --- | --- | --- | --- | --- | --- | --- | --- | --- | --- | --- |
| **M+0** | 0.5833 | 0.9159 | 0.4607 | 0.5373 | 0.5329 | 0.7873 | 0.5698 | 0.5782 | 0.6266 | 0.4452 | N/A*^a^* | 0.5917 | 0.5887 |
| **M+1** | 0.3894 | 0.0784 | 0.4101 | 0.2586 | 0.2925 | 0.2048 | 0.2958 | 0.3172 | 0.2831 | 0.3432 |  | 0.2517 | 0.2734 |
| **M+2** | 0.029 | 0.0057 | 0.1192 | 0.1439 | 0.1042 | 0.0154 | 0.1261 | 0.0847 | 0.0831 | 0.1728 |  | 0.1227 | 0.0909 |
| **M+3** | 0 |  | 0.0068 | 0.0683 | 0.0739 | 0 | 0.01 | 0.02 | 0.0051 | 0.0335 |  | 0.0295 | 0.0431 |
| **M+4** |  |  | 0.0036 | 0.0007 | 0.0069 |  | 0 | 0 | 0.0022 | 0.0053 |  | 0.0034 | 0.0084 |
| **M+5** |  |  | 0 | 0 | 0 |  | 0 |  |  | 0 |  | 0 | 0 |
| **M+6** |  |  |  | 0.0007 | 0.0005 |  |  |  |  |  |  | 0.001 | 0 |
| **M+7** |  |  |  |  |  |  |  |  |  |  |  | 0 | 0 |
| **M+8** |  |  |  |  |  |  |  |  |  |  |  | 0 | 0 |
| **M+9** |  |  |  |  |  |  |  |  |  |  |  | 0 | 0 |

*^a^* Peaks overlapped in GC/MS.

**(g)** 0.2% ^13^C-NaHCO_3_ and 0.1% unlabeled D-glucose

|  | **Ala** | **Gly** | **Val** | **Leu** | **Ile** | **Ser** | **Met** | **Thr** | **Asp** | **Glu** | **His** | **Phe** | **Tyr** |
| --- | --- | --- | --- | --- | --- | --- | --- | --- | --- | --- | --- | --- | --- |
| **M+0** | 0.9696 | 0.9723 | 0.9496 | 0.9183 | 0.9863 | 0.9722 | 0.8411 | 0.9373 | 0.9326 | 0.9449 | 0.921 | 0.9155 | 0.8951 |
| **M+1** | 0.0321 | 0.0264 | 0.0546 | 0.0793 | 0.0343 | 0.0323 | 0.154 | 0.0511 | 0.0671 | 0.0545 | 0.0743 | 0.0826 | 0.1024 |
| **M+2** | 0 | 0.0013 | 0 | 0 | 0 | 0 | 0.0551 | 0.0209 | 0.0016 | 0 | 0 | 0.0039 | 0 |
| **M+3** | 0 |  | 0 | 0.0266 | 0 | 0 | 0 | 0.0039 | 0 | 0 | 0.0033 | 0 | 0.0057 |
| **M+4** |  |  | 0 | 0 | 0.0013 |  | 0 | 0 | 0.0008 | 0 | 0 | 0 | 0.004 |
| **M+5** |  |  | 0 | 0 | 0.0009 |  | 0 |  |  | 0.0002 | 0 | 0 | 0 |
| **M+6** |  |  |  | 0.0005 | 0 |  |  |  |  |  | 0.0004 | 0.0001 | 0 |
| **M+7** |  |  |  |  |  |  |  |  |  |  |  | 0 | 0.0001 |
| **M+8** |  |  |  |  |  |  |  |  |  |  |  | 0 | 0.0001 |
| **M+9** |  |  |  |  |  |  |  |  |  |  |  | 0 | 0 |

**(h)** [3-^13^C]pyruvate

|  | **Ala** | **Gly** | **Val** | **Leu** | **Ile** | **Ser** | **Met** | **Thr** | **Asp** | **Glu** | **His** | **Phe** | **Tyr** |
| --- | --- | --- | --- | --- | --- | --- | --- | --- | --- | --- | --- | --- | --- |
| **M+0** | 0.0171 | 0.1988 | 0.0146 | 0 | 0 | 0.0208 | 0 | 0.0149 | 0.0126 | 0.0109 | 0.0098 | 0.0107 | 0.007 |
| **M+1** | 0.7182 | 0.5877 | 0.0207 | 0 | 0 | 0.3075 | 0 | 0.2059 | 0.1813 | 0.0154 | 0.0268 | 0.0026 | 0.0032 |
| **M+2** | 0.1695 | 0.2135 | 0.821 | 0.0981 | 0.0872 | 0.4777 | 0.1773 | 0.4048 | 0.3769 | 0.2857 | 0.082 | 0.0079 | 0.0112 |
| **M+3** | 0.0952 |  | 0.0773 | 0.7987 | 0.7212 | 0.194 | 0.4638 | 0.3738 | 0.4103 | 0.4502 | 0.2275 | 0.0635 | 0.0592 |
| **M+4** |  |  | 0.0496 | 0.0585 | 0.1201 |  | 0.3075 | 0.0006 | 0.0189 | 0.2261 | 0.3265 | 0.1728 | 0.1626 |
| **M+5** |  |  | 0.0169 | 0.0471 | 0.0674 |  | 0.0514 |  |  | 0.0118 | 0.2523 | 0.2604 | 0.2582 |
| **M+6** |  |  |  | 0 | 0.0041 |  |  |  |  |  | 0.0759 | 0.2437 | 0.2667 |
| **M+7** |  |  |  |  |  |  |  |  |  |  |  | 0.155 | 0.1449 |
| **M+8** |  |  |  |  |  |  |  |  |  |  |  | 0.0701 | 0.0831 |
| **M+9** |  |  |  |  |  |  |  |  |  |  |  | 0.0132 | 0.0039 |

**(i)** D-[6-^13^C]glucose

|  | **Ala** | **Gly** | **Val** | **Leu** | **Ile** | **Ser** | **Met** | **Thr** | **Asp** | **Glu** | **His** | **Phe** | **Tyr** |
| --- | --- | --- | --- | --- | --- | --- | --- | --- | --- | --- | --- | --- | --- |
| **M+0** | 0.4789 | 0.769 | 0.1835 | 0.1100 | 0.1340 | 0.2987 | 0.175 | 0.3021 | 0.2203 | 0.1371 | 0.0975 | 0.037 | 0.0368 |
| **M+1** | 0.4605 | 0.2022 | 0.4193 | 0.3021 | 0.3788 | 0.6092 | 0.3827 | 0.4597 | 0.4419 | 0.358 | 0.4136 | 0.1868 | 0.1971 |
| **M+2** | 0.0514 | 0.0288 | 0.3092 | 0.3988 | 0.3489 | 0.0783 | 0.3077 | 0.1964 | 0.2679 | 0.353 | 0.4056 | 0.3678 | 0.3712 |
| **M+3** | 0.0092 |  | 0.0682 | 0.1674 | 0.1098 | 0.0137 | 0.1245 | 0.0445 | 0.0655 | 0.1302 | 0.0543 | 0.3033 | 0.3004 |
| **M+4** |  |  | 0.0166 | 0.0124 | 0.0276 |  | 0.0159 | 0 | 0.0044 | 0.0206 | 0.0246 | 0.0757 | 0.0644 |
| **M+5** |  |  | 0.0031 | 0.0071 | 0.0078 |  | 0 |  |  | 0.0011 | 0.005 | 0.0224 | 0.0233 |
| **M+6** |  |  |  | 0.0023 | 0 |  |  |  |  |  | 0 | 0.006 | 0.0051 |
| **M+7** |  |  |  |  |  |  |  |  |  |  |  | 0.001 | 0.0009 |
| **M+8** |  |  |  |  |  |  |  |  |  |  |  | 0 | 0.0001 |
| **M+9** |  |  |  |  |  |  |  |  |  |  |  | 0 | 0.0006 |

**(j)** 25% D-[U-^13^C_6_]glucose/75% unlabeled D-glucose

|  | **Ala** | **Gly** | **Val** | **Leu** | **Ile** | **Ser** | **Met** | **Thr** | **Asp** | **Glu** | **His** | **Phe** | **Tyr** |
| --- | --- | --- | --- | --- | --- | --- | --- | --- | --- | --- | --- | --- | --- |
| **M+0** | 0.7301 | 0.7797 | 0.5643 | 0.4838 | 0.4563 | 0.7094 | 0.4734 | 0.5455 | 0.5712 | 0.4659 | 0.4779 | 0.4444 | 0.4198 |
| **M+1** | 0.1056 | 0.0935 | 0.1326 | 0.1426 | 0.1960 | 0.1206 | 0.2742 | 0.2223 | 0.2324 | 0.261 | 0.237 | 0.1446 | 0.1394 |
| **M+2** | 0.049 | 0.1268 | 0.1545 | 0.2605 | 0.2140 | 0.0727 | 0.1545 | 0.1538 | 0.1328 | 0.194 | 0.0952 | 0.1205 | 0.1293 |
| **M+3** | 0.1153 |  | 0.1118 | 0.0512 | 0.0822 | 0.0974 | 0.0736 | 0.0626 | 0.055 | 0.0603 | 0.093 | 0.1412 | 0.1475 |
| **M+4** |  |  | 0.0166 | 0.0547 | 0.0423 |  | 0.022 | 0.0158 | 0.0086 | 0.0165 | 0.0541 | 0.0771 | 0.0849 |
| **M+5** |  |  | 0.0201 | 0.0044 | 0.0112 |  | 0.0022 |  |  | 0.0024 | 0.035 | 0.0347 | 0.0399 |
| **M+6** |  |  |  | 0.0028 | 0 |  |  |  |  |  | 0.0077 | 0.0234 | 0.0216 |
| **M+7** |  |  |  |  |  |  |  |  |  |  |  | 0.0101 | 0.0115 |
| **M+8** |  |  |  |  |  |  |  |  |  |  |  | 0.0022 | 0.0031 |
| **M+9** |  |  |  |  |  |  |  |  |  |  |  | 0.0017 | 0.0029 |

**(k)** 100% D-[U-^13^C_6_]glucose

|  | **Ala** | **Gly** | **Val** | **Leu** | **Ile** | **Ser** | **Met** | **Thr** | **Asp** | **Glu** | **His** | **Phe** | **Tyr** |
| --- | --- | --- | --- | --- | --- | --- | --- | --- | --- | --- | --- | --- | --- |
| **M+0** | 0.0255 | 0.0673 | 0.0434 | 0.0440 | 0.0472 | 0.033 | 0.049 | 0.0347 | 0.0454 | 0.0173 | 0.0301 | 0.0739 | 0.0449 |
| **M+1** | 0.0048 | 0.0353 | 0.004 | 0.0041 | 0.0023 | 0.0099 | 0.016 | 0.0031 | 0.0037 | 0.0018 | 0.0042 | 0.0069 | 0.0062 |
| **M+2** | 0.0433 | 0.8974 | 0.0035 | 0.0114 | 0.0144 | 0.0458 | 0.0047 | 0.0078 | 0.0126 | 0.0036 | 0.0015 | 0.0004 | 0.0012 |
| **M+3** | 0.9265 |  | 0.0044 | 0.0040 | 0 | 0.9113 | 0.0159 | 0.1045 | 0.1071 | 0.0125 | 0.0023 | 0.0003 | 0.0008 |
| **M+4** |  |  | 0.0668 | 0.0130 | 0.0160 |  | 0.1322 | 0.8499 | 0.8311 | 0.1185 | 0.0069 | 0.0011 | 0.0024 |
| **M+5** |  |  | 0.878 | 0.1413 | 0.1429 |  | 0.7821 |  |  | 0.8463 | 0.0885 | 0.002 | 0.0014 |
| **M+6** |  |  |  | 0.7822 | 0.7814 |  |  |  |  |  | 0.8665 | 0.0014 | 0.0035 |
| **M+7** |  |  |  |  |  |  |  |  |  |  |  | 0.0085 | 0.0105 |
| **M+8** |  |  |  |  |  |  |  |  |  |  |  | 0.1024 | 0.1086 |
| **M+9** |  |  |  |  |  |  |  |  |  |  |  | 0.803 | 0.8206 |
