# Supplementary material for: Carbohydrate Metabolism and Carbon Fixation in Roseobacter denitrificans OCh114
Source: PLoS One. 2009 Oct 1;4(10):e7233. doi: 10.1371/journal.pone.0007233 (PMC2749216; doi:10.1371/journal.pone.0007233)
Supplement: Table S2 — Primers used for QRT-PCR studies reported in this paper (0.04 MB DOC) [file pone.0007233.s005.doc]

| **Gene name**  **(gene location)** | **Proteins** | **Forward primers** | **Reverse primers** |
| --- | --- | --- | --- |
| *16S rRNA* |  | TGTTCGGAATTACTGGGCG | TCGGGATTTCACCCCTAACTT |
| *pyc*  (RD1_3376) | pyruvate carboxylase | CCTTGGGCTTGCGGATC | CATCTGGTTCACCTCGGCA |
| *pckA*  (RD1_1376) | PEP carboxykinase | ATGCCATGCCAGTTCGGTA | CGGTGGTTCGGACCCC |
| *ppc*  (RD1_4248) | PEP carboxylase | GGCTCGATATTGCGGAACTC | ATCCTCTGCCATCGCCC |
| *tme*  (RD1_0421) | malic enzyme | (1)ACCCCCGGAAAGTTCGAG  (2)CGCCTATGATTACACCAACAAGG | (1)AAGACTGAGGTCCCGCTGC  (2)CCCAAGACCGCTGTCCC |
| *ppk*  (RD1_1948) | pyruvate phosphate dikinase | CAGGGTGTCTTTCCCGAAGA | AATCTGCAAAAGTTCCCCCA |
| *aatA*  (RD1_3892) | aspirate aminotrans-ferase | TCGAGCGGGCATAGGAAA | GGCTTGATTTTGACCCGGT |
| *eda*  (RD1_2878) | KDPG aldolase | CCAGAAGTGGTAATTCCAGCG | TTCACCCGGCGCGAC |
| *edd*  (RD1_2879) | phosphogluconate dehydrase | CGCACGGTGCTTTTTTCG | GTTCCTGCCAGCGGGTC |
| *ilvA*  (RD1_0416) | threonine deaminase | CACATGAAAGCGACGCCC | GCGTGATGTTTTTGTCTGCG |
