# Supplementary material for: Carbohydrate Metabolism and Carbon Fixation in Roseobacter denitrificans OCh114
Source: PLoS One. 2009 Oct 1;4(10):e7233. doi: 10.1371/journal.pone.0007233 (PMC2749216; doi:10.1371/journal.pone.0007233)
Supplement: Table S3 — The theoretical and experimental M+0 values in alanine and serine using D-[1-13C] glucose and D-[6-13C]glucosea,b (0.03 MB DOC) [file pone.0007233.s006.doc]

|  | **Theoretical (M+0)^Ala^ value** | **Experimental (M+0)^Ala^ value** | **Theoretical (M+0)^ser^ value** | **Experimental (M+0)^Ser^ value** |
| --- | --- | --- | --- | --- |
| **D-[1-^13^C]glucose** | 0.5 | 0.56 | 1.0 | 0.74 |
| **D-[6-^13^C]glucose** | 0.5 | 0.48 | 0 | 0.30 |

p.s.

*^a^* (M+0)^Ala^, M+0 value for alanine; (M+0)^Ser^, M+0 value for serine

*^b^* The theoretical (M+0)^Ala^ and (M+0)^Ser^ values were estimated by the ED pathway.

Estimate the contribution of the PP pathway for carbohydrate metabolism. The difference between the theoretical and experimental (M+0) values in serine can be considered the contribution of the non-oxidative PP pathway, and approximately 25-30% GAP is estimated to be produced by the non-oxidative PP pathway. The difference between the theoretical and experimental M+0 values in alanine, 2-6%, can be considered as the experimental error.
